# Induction of Recombinant Lectin Expression by an Artificially Constructed Tandem Repeat Structure: A Case Study Using *Bryopsis plumosa* Mannose-Binding Lectin

**DOI:** 10.3390/biom8040146

**Published:** 2018-11-14

**Authors:** Hyun-Ju Hwang, Jin-Woo Han, Hancheol Jeon, Jong Won Han

**Affiliations:** Department of Genetic Resources, National Marine Biodiversity Institute of Korea, Seocheon 33662, Korea; hjhwang@mabik.re.kr (H.-J.H.); hiclow@mabik.re.kr (J.-W.H.); hjeon@mabik.re.kr (H.J.)

**Keywords:** *Bryopsis plumosa*, BPL2, lectin, hemagglutinin, recombinant, tandem repeat

## Abstract

Lectin is an important protein in medical and pharmacological applications. Impurities in lectin derived from natural sources and the generation of inactive proteins by recombinant technology are major obstacles for the use of lectins. Expressing recombinant lectin with a tandem repeat structure can potentially overcome these problems, but few studies have systematically examined this possibility. This was investigated in the present study using three distinct forms of recombinant mannose-binding lectin from *Bryopsis plumosa* (BPL2)—i.e., the monomer (rD1BPL2), as well as the dimer (rD2BPL2), and tetramer (rD4BPL2) arranged as tandem repeats. The concentration of the inducer molecule isopropyl β-D-1-thiogalactopyranoside and the induction time had no effect on the efficiency of the expression of each construct. Of the tested constructs, only rD4BPL2 showed hemagglutination activity towards horse erythrocytes; the activity of towards the former was 64 times higher than that of native BPL2. Recombinant and native BPL2 showed differences in carbohydrate specificity; the activity of rD4BPL2 was inhibited by the glycoprotein fetuin, whereas that of native BPL2 was also inhibited by d-mannose. Our results indicate that expression as tandem repeat sequences can increase the efficiency of lectin production on a large scale using a bacterial expression system.

## 1. Introduction

Lectins are antibody-like proteins that specifically bind to carbohydrates [1] via two or more sugar-binding sites [2]. Lectins can agglutinate erythrocytes by binding the surface carbohydrates of red blood cells [1], and they are thus also referred to as agglutinin or hemagglutinin. Owing to this property, lectins can serve as pharmacological agents and as a diagnostic and therapeutic tool [3,4,5]. For example, they are used in combination with fluorophores to characterize cell-surface carbohydrates [6]. Lectin-conjugated resins for carbohydrate or glycoprotein isolation are commercially available (Vector laboratories, Burlingame, CA, USA). Several lectins have been suggested as candidate cancer diagnostic reagents [7], and lectin arrays have been used to determine carbohydrate structures or amino acid sequences [8]. Based on the myriad functions of lectins, the term functional lectinomics has been coined to refer to the sugar code of proteins [9,10]. Lectins have also been used in combination with a microarray approach to analyze the bacterial glycome [11].

However, the widespread application of lectins is hindered by several issues. Firstly, lectins from natural sources are often purified with other lectin isoforms [12], and they can therefore have unintended effects [13]. Secondly, it is difficult to obtain lectins in sufficient amounts from natural sources for industrial applications. Producing active proteins by recombinant technology can overcome these problems [14], and some plant and animal lectins have been successfully generated using a bacterial expression system [14,15,16]. However, the unexpected folding of recombinant lectins can lead to a loss of activity. For this reason, few recombinant marine algal lectins have been reported to date [12].

**Figure 1:**
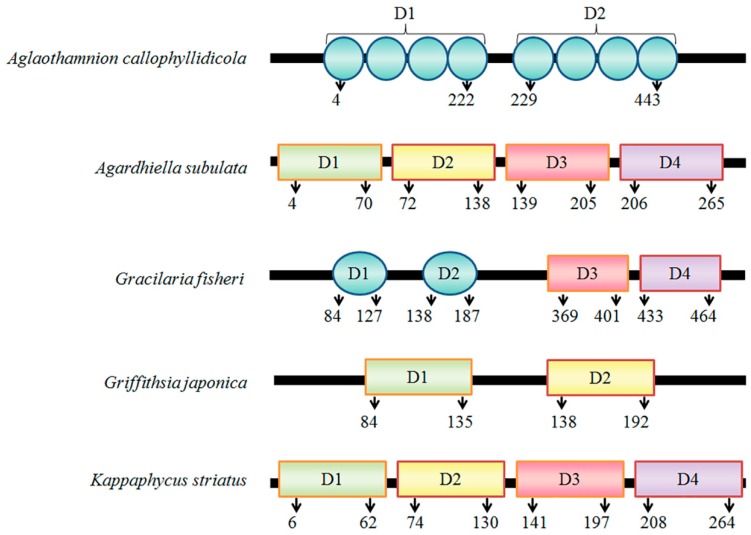
Primary structure of red algal lectins with a tandem repeat structure. The sequences were obtained from NCBI GenBank under the following accession numbers: *A. callophyllidicola* (from Shim et al. [18]), *A. subulata* lectin (ASL-1, BAX08598), *G. fisheri* or *H. fisheri* lectin (ACY56710), *G. japonica* glycoprotein (AAM93989), and *K. striatus* lectin (KSA2, BAR91206).

We recently showed that tandem repeat structures contribute to the expression of active red algal lectins [12,13]. Approximately 45 red algal lectins have been purified, of which only seven have been cloned and identified [17]. Based on the few reported sequences, we determined that red algal lectins contain tandem repeats, and that the active protein can be successfully expressed in bacterial cells (Figure 1). For example, rhodobindin-a lectin produced by the red alga *Aglaothamnion callophyllidicola*-contains an internal tandem repeat structure with at least eight domains [18], and *Gracilaria fisheri*, *Griffithsia japonica*, and *Kappaphycus striatus* lectins have sequentially arranged conserved domains. These observations imply that the tandem repeat structure contributes to the production of an active protein in recombinant expression systems. However, this has only been investigated for *N*-acetyl-D-glucosamine- and *N*-acetyl-D-galactosamine-specific lectins from *Bryopsis plumosa* (BPL3) [12]; more studies are required using different types of lectin lacking a tandem repeat and showing no activity in a bacterial expression system, in order to determine whether this modification can actually increase recombinant protein production.

Mannose-binding lectins function as anti-viral agents by binding to viral capsid mannan residues to inhibit viral infection [16,19]. BPL2 is produced by *B. plumosa*, and it differs from other *Bryopsis* lectins in terms of carbohydrate specificity [20]. The present study investigated whether arranging the BPL2 sequence as tandem repeats improves the expression of the recombinant protein, and established the optimal conditions for protein expression.

## 2. Materials and Methods

### 2.1. Preparation of Native BPL2

*B. plumosa* was cultured at 20 °C on a 12:12 h light/dark cycle in IMR medium. Plant materials were removed, and the culture medium was replaced every week. BPL2 was purified according to a previous study [20]. Crude extract was prepared in five volumes of phosphate-buffered saline (PBS, pH 7.2). Cell debris was removed by centrifugation at 40,000 *g* for 30 min at 4 °C. The extract was loaded on a mannose–agarose column, and BPL2 was eluted with 0.5 M mannose in 1 × PBS. The fractions containing BPL2 were pooled and stored at −80 °C until use.

### 2.2. Cloning and Construction of Recombinant BPL2

BPL2 cDNA was codon-optimized for bacteria K-12 codons using Genious program v.8.1. cDNA was synthesized by Bioneer (Daejeon, Korea) and cloned into the pBHA vector. The cDNA for three different forms of BPL2 was prepared-i.e., the monomer (rD1BPL2), dimer (rD2BPL2), and tetramer (rD4BPL2), with the latter two generated by joining the monomer sequences with internal spacers. Synthesized constructs were digested with the restriction enzyme combinations *Bam*HI/*Sac*I (rD1BPL2), *Bam*HI/*Sal*I (rD2BPL2), and *Bam*HI/*Xho*I (rD4BPL2) at 37 °C for 1 h, and they were then inserted into the pET28a(+) vector (Invitrogen, Carlsbad, CA, USA) by incubation with 4 U T4 DNA ligase for 12 h at 12 °C. Plasmids were transformed into *Escherichia coli* DH5α cells, which were cultured overnight at 37 °C. The plasmids were purified and the sequences were confirmed by sequencing on an ABI 3730 DNA system (Applied Biosystems, Foster City, CA, USA).

*E. coli* BL21(λDE3) cells (Invitrogen) were transformed by *pET28a: BPL2*. The transformants were spread on Luria-Bertani (LB) agar plates containing 25 μg/mL kanamycin. Positive colonies were isolated and subcultured in 10 mL LB-kanamycin medium.

### 2.3. Optimization of rBPL2 Expression

Transformants were subcultured overnight at 37 °C in LB medium containing 25 μg/mL kanamycin. A 1 mL volume was inoculated in 100 mL LB medium in an Erlenmeyer flask, followed by incubation on a shaker until the optical density at 600 nm (OD_600_) was 0.4–0.6; 10 mL of the sample was collected from the flask as the uninduced control.

To determine the optimum concentration of isopropyl β-D-1-thiogalactopyranoside (IPTG) for the induction of recombinant protein expression, bacterial cultures (OD 0.4–0.6) were treated with various concentrations of IPTG (0.1, 0.2, 0.4, 0.5, and 1 mM) at 37 °C for 3 h. To determine the optimum temperature, 0.5 mM IPTG was added to the culture (OD 0.4–0.6) at various temperatures (20 °C, 25 °C, 30 °C, and 37 °C) for 3 h. The induction efficiency was determined at various times after adding IPTG. Samples of 10 mL were collected under different conditions, and cell pellets were obtained by centrifugation at 20,000 *g* for 10 min.

Total protein from each sample was extracted by heating at 90 °C for 5 min in 1 × sodium dodecyl sulfate-polyacrylamide gel electrophoresis (SDS-PAGE) sample buffer (0.2 mL/mL culture). The samples were cooled to room temperature and centrifuged to obtain the supernatant as a crude extract. The expression level of each construct was evaluated by SDS-PAGE. Soluble fractions were prepared from the cell pellet (10 mL) by sonication in 1 mL extraction buffer composed of 1 mM phenylmethylsulfonyl fluoride in 1× PBS (pH 7.2) at 15% amplitude, with 15 s/5 s on/off periods repeated 20 times. The supernatant was collected by centrifugation at 20,000 *g* for 10 min, and expression efficiency was calculated by measuring the protein band intensity on the gel with GelAnalyzer 2010 (Lazar Software, Debrecen, Hungary).

### 2.4. Purification of rBPL2

Bacterial cultures were harvested by centrifugation at 5000 *g* for 10 min. Cell pellets were resuspended in urea extraction buffer composed of 50 mM NaH_2_PO_4_, 300 mM NaCl, and 8 M urea (pH 8.0). Cell extracts were prepared by sonication at 15% amplitude, with 3 s on/off periods repeated 20 times. Bacterial cell debris was removed by centrifugation at 20,000 *g* for 20 min at 4 °C. The supernatant was collected as the crude extract, which was purified by nickel–nitrilotriacetic acid (Ni-NTA) chromatography using a fast protein liquid chromatography system (Bio-Rad, Hercules, CA, USA). The column was washed with 10 volumes of wash buffer composed of 50 mM NaH_2_PO_4_, 300 mM NaCl, 8 M urea, and 25 mM imidazole (pH 8.0) at a flow rate of 1 mL/min. Recombinant protein was eluted with an imidazole step gradient (10 volumes of 75, 125, and 250 mM imidazole in extraction buffer). The eluent was monitored with an ultraviolet–visible light detector at 245, 280, and 360 nm. The fractions were analyzed by SDS-PAGE, and those containing rBPL2 were collected and pooled.

### 2.5. Refolding of rBPL2

The recombinant protein produced as an inclusion body was refolded in refolding buffer (0.3 M NaCl in 1 × PBS (pH 7.5)) until the urea concentration in the protein solution reached 4 M. Purified protein was passed through a 0.45-µm syringe filter to remove any nuclei that could cause protein crystallization. The refolding buffer was slowly added to the protein solution using the BioLogic LP system (Bio-Rad) at a flow rate of 0.05 mL/min at room temperature with continuous stirring. The protein solution was incubated at room temperature for 3 h and then centrifuged at 20,000 *g* for 10 min to remove insoluble materials. The soluble fraction was recovered as the refolded recombinant protein fraction.

### 2.6. Hemagglutination Activity Assay

Hemagglutination activity was evaluated as previously described [21]. Horse blood was obtained from HanilComed (Seongnam, Korea); the blood cells were washed with PBS until the supernatant no longer had a red color. A 4% erythrocyte suspension was prepared in PBS. Samples were prepared by serial two-fold dilution in a final volume of 25 µL in U-bottomed microplates. The erythrocyte suspension was added to each well, followed by incubation for 30 min at room temperature. The minimum amount of lectin required for complete agglutination was defined as 1 hemagglutination unit (HU).

### 2.7. Analysis of Carbohydrate Specificity

Carbohydrate specificity was evaluated based on the inhibition of hemagglutination activity [21]. Serial two-fold dilutions of carbohydrate samples were prepared in PBS and mixed with an equal volume of 4 HU recombinant lectin. *N*-acetyl-glucosamine, *N*-acetyl-galactosamine, L-fucose, D-galactose, D-glucose, D-mannose, D-fructose, and lactose at 500 mM or glycoprotein, fetuin, and asialofetuin at 100 mg/mL were used for the inhibition test. After combining the carbohydrate and recombinant lectin, an equal volume (25 µL) of a 4% horse erythrocyte suspension was added to the mixture. The minimum inhibitory concentration of sugar in the final reaction mixture was determined.

### 2.8. Glycan Microarray

A glycan microarray analysis was performed using the Glycan-300 array kit (RayBioTech, Norcross, GA, USA), which consisted of 300 synthetic glycans printed in quadruplicate on a glass slide. The experiment was performed by Ebiogen (Seoul, Korea), according to a previously published protocol (Hwang et al. 2018). Biotinylated recombinant lectin (rD4BPL2) and native BPL2 at 100 μg/mL were added to the array wells. The slide was incubated for 3 h with gentle agitation. The glass slide was washed with 1 × wash buffers I and II provided in the kit. Cy3-equivalent dye-conjugated streptavidin was used for glycan-lectin binding. The signals were visualized using a microarray laser scanner (Genfix 4100A; Molecular Devices, Sunnyvale, CA, USA) with excitation at 554 nm and emission at 568 nm. Data extraction was performed using Genfix microarray analysis software. Glycan array data were normalized and analyzed using RayBio Analysis software (RayBioTech, Norcross, GA, USA).

## 3. Results

### 3.1. Cloning of rBPL2

**Figure 2:**
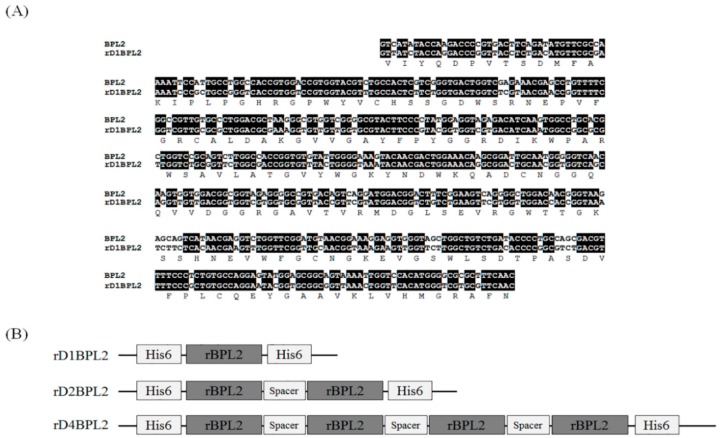
Construction of different forms of recombinant BPL2 by tandem repeats. (**A**) Codon optimization of cDNA and deduced amino acid sequence. (**B**) Illustration of recombinant protein structure, in which have tandem repeats were connected by spacer sequences. All constructs contained a His tag to allow for isolation/purification.

**Figure 3:**
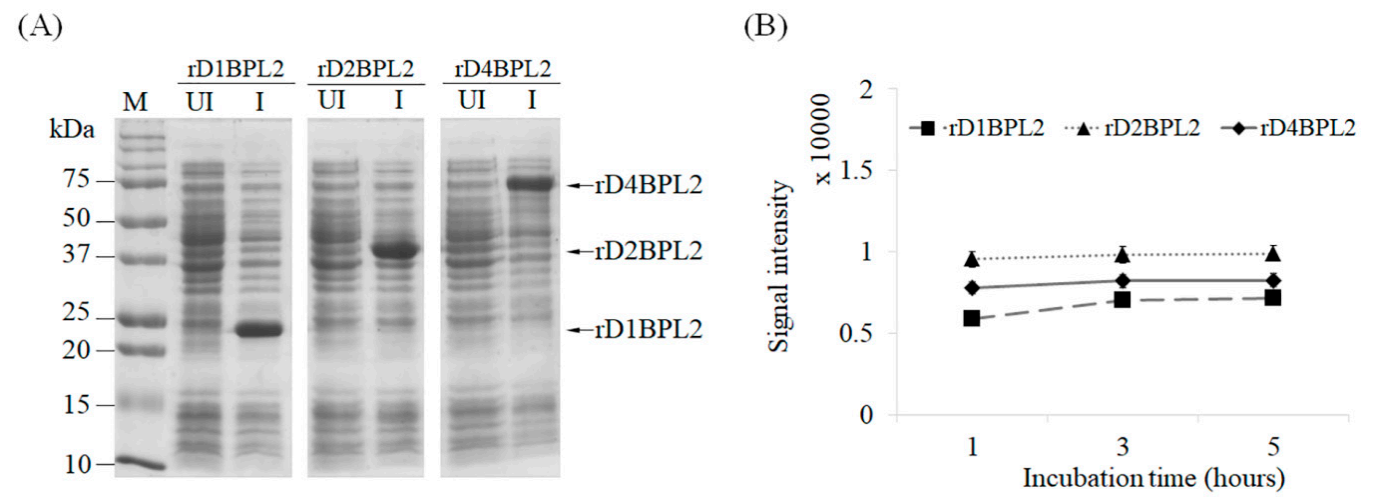
Expression of rBPL2 (Recombinant *Bryopsis plumosa* lectin 2). rD1BPL2 (monomeric), rD2BPL2 (dimeric), and rD4BPL2 (tetrameric) expressed in *E. coli* BL21 (λDE3). (**A**) M, Molecular weight marker; UI, uninduced lysate; I, induction with isopropyl β-D-1-thiogalactopyranoside (IPTG) for 5 h. Arrows indicate target proteins. (**B**) Expression level of recombinant proteins at different induction times. Squares, rD1BPL2; triangles, rD2BPL2; diamonds, rD4BPL2.

BPL2 was codon-optimized for bacterial codons (Figure 2A). To assess the effect of the repeated sequence on lectin expression and activity, we prepared three different constructs: the monomer, which was the original form of BPL2; and dimeric and tetrameric forms containing two and four BPL2 sequences, respectively (Figure 2B). Although there were slight differences, the expression efficiency was mostly similar for all constructs (Figure 3A) and was unaffected by incubation time after addition of the inducer IPTG (Figure 3B).

### 3.2. Optimization of Expression Conditions

**Figure 4:**
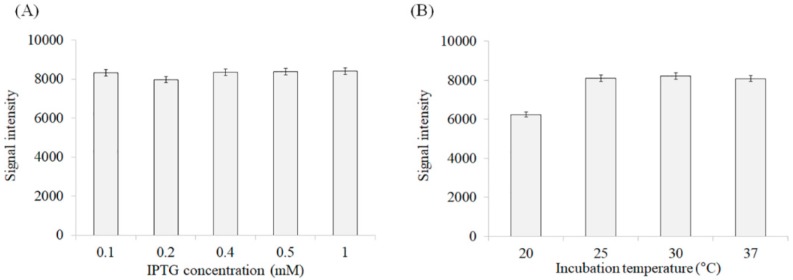
Expression efficiency of rD4BPL2 (Recombinant *Bryopsis plumosa* lectin 2, tetrameric construction) under different conditions. (**A**) Induction efficiency at different isopropyl β-D-1-thiogalactopyranoside (IPTG) concentrations (at 37 °C, OD600 = 0.6–0.8 for 3 h), (**B**) Effect of temperature (20–37 °C) on the efficiency of recombinant protein induction (OD600 = 0.6–0.8, 0.5 mM IPTG for 3 h).

The expression efficiency was measured at different IPTG concentrations (0.1–1 mM), and at different incubation temperatures (20–37 °C). The efficiency of recombinant protein expression was independent of IPTG concentration and incubation time, since the amount of protein induced was similar under all tested conditions (Figure 4A). Meanwhile, temperature had a slight effect on the expression efficiency, with a higher rate being observed at temperatures over 25 °C (Figure 4B).

### 3.3. Purification of Recombinant Lectins

**Figure 5:**
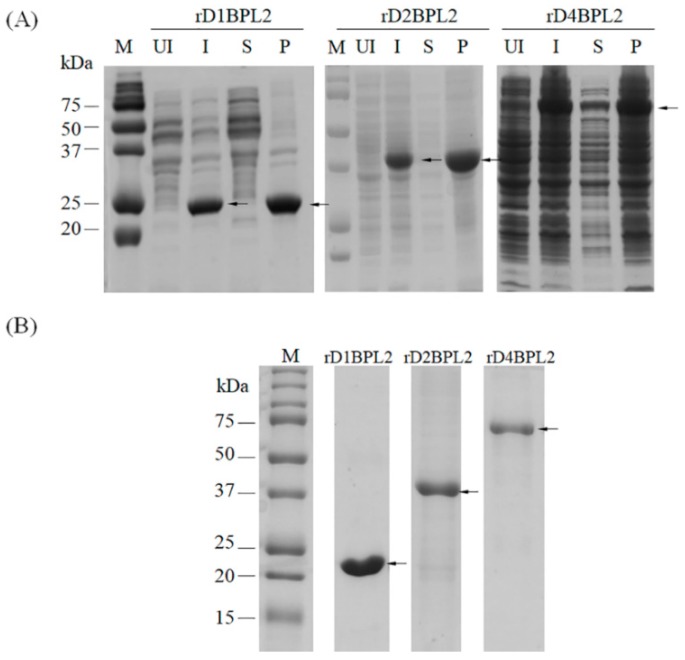
Solubility analysis and purification of recombinant lectins by Ni-NTA agarose chromatography. (**A**) Solubility of recombinant proteins. UI, total protein of the un-induced cell fraction; I, induced; S, soluble in PBS; P, pellet. Arrows indicate target proteins. (**B**) Purified recombinant lectins. M, molecular weight marker. Arrows indicate purified recombinant proteins.

The three recombinant lectins were purified by Ni–NTA affinity chromatography. None of the lectins were extracted in PBS in a soluble form (Figure 5A). Extracts were solubilized and prepared in denaturing solution (8 M urea in PBS). Most of the lectins were bound to the affinity resin. A gradient of 75, 125, and 250 mM imidazole was used to elute the recombinant proteins, yielding a single protein band (Figure 5B). The peptide sequence of the protein was matched to translated cDNA sequences (data not shown).

### 3.4. Hemagglutination Activity of Recombinant Lectins

**Figure 6:**
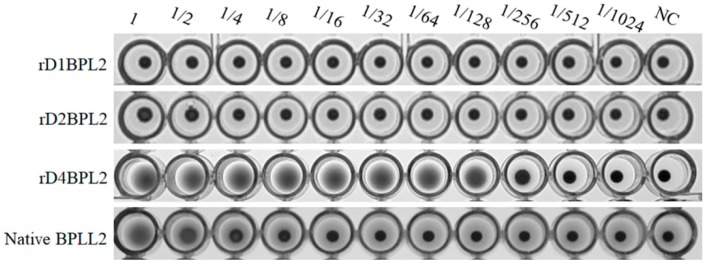
Hemagglutination activity of recombinant and native BPL2. Serial two-fold dilutions are shown from left to right. NC, negative control. The protein concentration in the first well was 550 μg/mL for the recombinant and 160 μg/mL for native BPL2.

The monomeric and dimeric lectins did not agglutinate horse erythrocytes at any concentration, but the tetramer did (Figure 6 and Table 1). The activity of rD4BPL2 was about 64 times higher than that of native BPL2 towards horse erythrocytes, but weaker than that of native BPL2 towards sheep erythrocytes (Table 1). The minimum concentration of recombinant protein required for agglutination of horse erythrocytes was 4.2 μg/mL, as compared to 160 μg/mL for native BPL2 (Figure 6).

### 3.5. Carbohydrate Specificity

rD4BPL2 was not inhibited by d-mannose, which is a carbohydrate that is complementary to native BPL2. The tested mono- and disaccharides did not inhibit the agglutination activity of rD4BPL2, which was suppressed only by treatment with the glycoprotein bovine serum fetuin. The minimum concentration of fetuin required for inhibition was 12.2 μg/mL (Table 2).

To establish the carbohydrate specificity of BPL2, we carried out a glycan microarray. Only native BPL2, and not the recombinant protein, could be analyzed. The major carbohydrates targeted by native BPL2 were maltohexaose-β-Sp1 and maltoheptaose-β-Sp1, although weak signals were observed for the monosaccharides α-Man-Sp, β-Glc-Sp, and β-Gal-Sp (Supplementary Table S1).

## 4. Discussion

Lectin with a tandem repeat structure has been reported in wide range of organisms, including catfish egg [22], ponyfish [23], and *Eucheuma serra* [24]. Griffithsin tandemers in engineered tandem repeats of mGRFT were shown to exhibit antiviral activity [25].

BPL2, a mannose-binding lectin from *B. plumosa*, is well-suited to investigations of engineered tandemers, since it has no repeat domains in its sequence. SDS-PAGE, size-exclusion chromatography, and mass spectrometry findings suggest that native BPL2 exists as a monomer in nature [20], although an active form of the protein has yet to be generated using a bacterial expression system. BML-17, a mannose-binding lectin from *Bryopsis maxima*, may have inhibitory activity against human immunodeficiency virus [26], although this not been confirmed due to the difficulty in obtaining a sufficient amount of *Bryopsis* sp. for testing.

In order to clarify the contribution of tandem repeats to lectin activity, and produce recombinant BPL2, we expressed three forms of the protein with different numbers of tandem repeats from the pET28a vector. All three proteins (rD1BPL2, rD2BPL2, and rD4BPL2) were successfully expressed with similar efficiencies in the *E. coli* expression system. A previous study reported that the dimeric form of the *N*-acetyl-d-glucosamine- and *N*-acetyl-galactosamine-specific lectin rBPL3 was more highly expressed than the monomer [12]. The efficient expression of foreign genes in bacteria depends on various factors, including the amino acid composition and the arrangement of protein domains [27]. The results of the present study suggest that the tandem repeat structure does not affect the efficiency of the recombinant BPL2 expression. Since the IPTG concentration and induction time also had no effect, these factors were not considered in subsequent experiments.

The solubility of rBPL2 was not increased by the tandem repeat structure; none of the proteins were soluble in PBS, and they were only solubilized under the denaturing conditions required for refolding. The proteins were precipitated when the urea concentration was 3 M. In contrast, the solubility of rRhodobindin was improved by increasing the number of domain repeats [13]. The solubility of recombinant proteins is affected by factors such as *E. coli* strain, culture temperature, and induction method [28]. Repeated domains are considered as one factor affecting solubility, but not necessarily in an expression system. Given the lack of solubility of the recombinant proteins in this study, other experiments, including the hemagglutination activity assay, were performed in the presence of 4 M urea. The conditions that can increase the solubility of rBPL2 remain to be determined.

Many red algal lectins (e.g., *Solieria filiformis* lectin [29]) have four to eight tandem repeats of short domains or motifs. The function of these repeated sequences is unclear; prediction algorithms have been developed to clarify their role in protein function and their evolutionary significance [30]. It is possible that tandem repeats provide multiple binding sites and stabilize the structure of a protein [31]. Accordingly, proteins have been designed to include tandem arrays of a repeated structural motif [30,32]. Many plant lectins contain a single domain or are encoded by multiple interspersed copies of the corresponding gene, requiring assembly to generate a functional protein (e.g., concanavalin A). Red algal lectins may be more efficiently expressed in prokaryote expression systems, since they do not require this type of assembly.

rD4BPL2 showed strong hemagglutination activity against erythrocytes while the other forms of the protein (rD1BPL2 and rD2BPL2) did not. Interestingly, the hemagglutination activity towards horse erythrocytes was higher than that of native BPL2 isolated from a natural source, suggesting that the tandem repeat structure enhances protein activity, although the precise mechanism remains unclear. Similarly, mGRFT composed of a tandem array of GRFT sequences exhibited potent antiviral activity, as compared to the native or monomeric proteins [25]. Another study reported that only the dimeric form of BPL3 had hemagglutination activity [12]. Reducing the number of domain repeats also altered rRhodobindin hemagglutination activity and solubility [13]. Three-dimensional (3D) structure predictions suggest that the linear structure of rD2BPL2 reduces protein activity relative to rD4BPL2, although further studies are needed to confirm this possibility.

rBPL2 showed strong agglutination activity towards horse erythrocytes at a low concentration. The opposite was true for native BPL2. This may due to the different 3D structures of recombinant and native BPL2 caused by protein misfolding. Horse and sheep erythrocytes also have distinct cell surface oligosaccharides [33,34], which could explain the difference in carbohydrate specificity between rD4BPL2 and native BPL2.

As expected, native BPL2 showed specificity for d-mannose and bovine serum fetuin, whereas rD4BPL2 activity was not inhibited by the tested monosaccharides, including d-mannose, but it was suppressed by the glycoprotein bovine serum fetuin. This differs from the previous finding that recombinant and native BPL3 showed similar sugar specificities, but different affinities for α and β amino sugars, which was attributed to misfolding that can alter the properties of a protein [12].

A glycan microarray was performed to evaluate the sugar specificity of rD4BPL2, but we were unable to obtain results due to the high concentration of the denaturant urea. Native BPL2 showed a high signal intensity upon binding to maltohexaose-β-Sp1 and maltoheptaose-β-Sp1, which are high molecular weight glucose chains. Native BPL2 also bound-albeit weakly-to the monosaccharides α-Man-Sp, β-Glc-Sp, and β-Gal-Sp. Maltose did not inhibit the hemagglutination activity of BPL2; this may require a polymer that includes at least six glucose molecules. It is possible that recombinant proteins bind to oligosaccharides such as maltohexaose based on their glycoprotein binding properties.

## 5. Conclusions

An active form of rBPL2 was successfully generated from a construct with artificial tandem repeats of the lectin sequence. Neither the monomer nor the dimer showed hemagglutination activity. However, the tetramer potently induced hemagglutination of erythrocytes to a degree that was comparable to native BPL2. These results suggest that tandem repeats can enhance lectin activity. Recombinant and native BPL2 showed differences in carbohydrate specificity that must be overcome for the recombinant protein to be used in industrial applications. Nonetheless, tandem repeat sequences are a useful tool for the large-scale production of lectins with high hemagglutination activity using a bacterial expression system.

## Figures and Tables

**Table 1:** Minimum lectin concentration for agglutination of a 4% erythrocyte suspension.

Protein	Horse Erythrocytes	Sheep Erythrocytes
rD1BPL2	ND	ND
rD2BPL2	ND	ND
rD4BPL2	4.2 μg/mL	ND
Native BPL2	160 μg/mL	0.3 μg/mL

ND, no hemagglutination at 550 μg/mL.

**Table 2:** Minimum concentrations of various substances for lectin inhibition.

Substance	Minimum Inhibitory Concentration	
	rD4BPL2	Native BPL2
d-Glucose	NI	NI
d-Galactose	NI	NI
d-Mannose	NI	62.5 mM
N-Acetyl-d-glucosamine	NI	NI
N-Acetyl-d-galactosamine	NI	NI
l-fucose	NI	NI
Maltose	NI	NI
Lactose	NI	NI
Fructose	NI	NI
Fetuin	12.2 μg/mL	195.3 μg/mL

NI, no inhibition at 500 mM.

## Supplementary Materials

The following are available online at http://www.mdpi.com/2218-273X/8/4/146/s1: Figure S1, Predicted 3-dimensional structure of BPL2; Table S1, Glycan microarray of native BPL2.
