# Supplementary material for: Induction of Recombinant Lectin Expression by an Artificially Constructed Tandem Repeat Structure: A Case Study Using Bryopsis plumosa Mannose-Binding Lectin
Source: Biomolecules. 2018 Nov 14;8(4):146. doi: 10.3390/biom8040146 (PMC6316659; doi:10.3390/biom8040146)
Supplement: Supplementary file 1 [file biomolecules-08-00146-s001.pdf]

Supplementary files

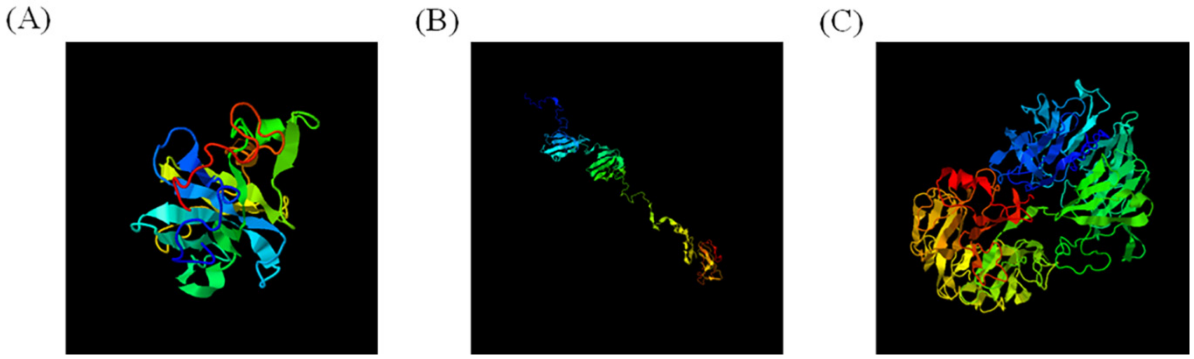

Figure S1. Predicted three-dimensional structure of BPL2. The structure was determined by protein sequence similarity analysis using the I-TASSER program (https://zhanglab.ccmb.med.umich.edu/I-TASSER/) with normal parameters; a higher C score indicates a model with a higher confidence. (A) Native BPL2, C score = −2.64. (B) rD2BPL2, C score = −2.86. (C) rD4BPL2.

Table S1. Glycan microarray of native BPL2, with binding signals normalized using a program provided by RayBioTech

Substrate	Glycan structure	Normalized RFU	
		Mean	SD
**POS1**	–	5687	0
**NEG**	-	370	25
**G0001**	β-Glc-Sp	1785	853
**G0002**	β-Gal-Sp	1708	519
**G0003**	α-Man-Sp	1794	461
**G0004**	α-Fuc-Sp	270	17
**G0005**	α-Rha-Sp	227	46
**G0006**	β-GlcNAc-Sp	814	201
**G0007**	β-GalNAc-Sp	616	120
**G0008**	Tobramycin	823	189
**G0009**	Gal-β-1,3-GlcNAc-β-Sp	363	71
**G0010**	Gal-α-1,3-Gal-β-1,3-GlcNAc-β-Sp	212	57
**G0011**	Neu5Ac-α-2,3-Gal-β-1,3-GlcNAc-β-Sp	452	125
**G0012**	Neu5Ac-α-2,6-Gal-β-1,3-GlcNAc-β-Sp	409	117
**G0013**	Neu5Gc-α-2,3-Gal-β-1,3-GlcNAc-β-Sp	381	78
**G0014**	Neu5Gc-α-2,6-Gal-β-1,3-GlcNAc-β-Sp	422	115
**G0015**	Gal-β-1,3-(Fuc-α-1,4)-GlcNAc-β-[Lewis A]-Sp	394	35
**G0016**	Gal-β-1,4-Glc-β-Sp	905	162
**G0017**	Gal-α-1,3-Gal-β-1,4-Glc-β-Sp	251	95
**G0018**	Gal-α-1,4-Gal-β-1,4-Glc-β-Sp	846	205
**G0019**	GlcNAc-β-1,3-Gal-β-1,4-Glc-β-Sp	519	72
**G0020**	GalNAc-β-1,3-Gal-β-1,4-Glc-β-Sp	731	272
**G0021**	Neu5Ac-α-2,3-Gal-β-1,4-Glc-β-Sp	198	28

**G0022**	Neu5Ac-α-2,6-Gal-β-1,4-Glc-β-Sp	439	129
**G0023**	Neu5Gc-α-2,3-Gal-β-1,4-Glc-β-Sp	425	175
**G0024**	Neu5Ac-α-2,6-Gal-β-1,4-Glc-β-Sp	484	183
**G0025**	Gal-β-1,4-(Fuc-α-1,3)-Glc-β-Sp	498	37
**G0026**	GalNAc-β-1,3-Gal-α-1,4-Gal-β-1,4-Glc-β-Sp	146	12
**G0027**	GlcNAc-β-1,6-GlcNAc-β-Sp	432	11
**G0028**	4-P-GlcNAc-b-1,4-Man-b-Sp	350	27
**G0029**	Glc-α-1,2-Gal-α-1,3-Glc-α-Sp	359	121
**G0030**	Gal-β-1,3-GalNAc-α-Sp	295	53
**G0031**	Gal-β-1,4-GlcNAc-β-Sp	562	149
**G0032**	Gal-β-1,4-(Fuc-α-1,3)-GlcNAc-β-[Lewis X]–Sp	449	152
**G0033**	Neu5Ac-α-2,3-Gal-β-1,4-(Fuc-α-1,3)-GlcNAc-β-[sialyl Lewis X]-Sp	329	63
**G0034**	Neu5Ac-α-2,3-Gal-β-1,3-(Fuc-α-1,4)-GlcNAc-β-[sialyl Lewis A]-Sp	202	93
**G0035**	Neu5Gc-α-2,3-Gal-β-1,3-(Fuc-α-1,4)-GlcNAc-β-[sialyl Lewis A]-Sp	156	34
**G0036**	Gal-α-1,4-Gal-β-1,3-GlcNAc-β-Sp	299	61
**G0037**	Gal-β-1,4-GlcNAc-β-1,3-Gal-β-1,4-Glc-β-[LNnT]-Sp	254	68
**G0038**	GlcA-β-1,4-GlcNAc-α-1,4-GlcA-β-Sp	192	66
**G0039**	GlcNAc-β-1,6-(Gal-β-1,3)-GalNAc-α-O-Ser-Sp4	15	6
**G0040**	Neu5Ac-α-2,3Gal-β-1,4-(6S)GlcNAc-β-Sp	331	43
**G0041**	GalNAc-β-1,4-GlcNAc-β-Sp2	379	41
**G0042**	Neu5Ac-α-2,8-Neu5Ac-α-2,3-Gal β-1,4-Glc-β-Sp	85	23
**G0043**	Neu5Gc-α-2,8-Neu5Ac-α-2,3-Gal-β-1,4-Glc-β-Sp	219	48
**G0044**	GalNAc-α-1,3-(Fuc-α-1,2)-Gal-β-1,4-Glc-β-[blood A antigen tetrose]-Sp1	114	1
**G0045**	GlcNAc-β-1,2-Man-α-Sp	146	2
**G0046**	Neu5Ac-α-2,3-Gal-β-Sp1	215	22
**G0047**	Gal-β-1,3-GalNAc-β-1,3-Gal-β-Sp1	102	25
**G0048**	Glc-α-1,2-Gal-α-Sp	767	172

**G0049**	Gal-β-1,4-(Fuc-α-1,3)-GlcNAc-β-1,3-Gal-β-Sp1	281	29
**G0050**	Neu5Ac-α-2,3-Gal-β-1,4-(Fuc-α-1,3)-Glc-β-[3-sialyl-3-fucosyllactose/F-SL]-Sp1	173	70
**G0051**	GlcNAc-β-1,4-GlcNAc-β-Sp1	410	142
**G0052**	β-D-GlcA-Sp	736	276
**G0053**	Gal-β-1,4-(6S)GlNAc-β-Sp	424	94
**G0054**	GlcNAc-α-1,3-(Glc-α-1,2-Glc-α-1,2)-Gal-α-1,3-Glc-α-Sp	124	53
**G0055**	Gal-β-1,3-GalNAc-β-1,4-(Neu5Gc-α-2,3)-Gal-β-1,4-Glc-β-Sp1	173	16
**G0056**	Sisomicin sulfate	1652	370
**G0057**	GalNAc-α-1,3-(Fuc-α-1,2)-Gal-β-[blood A antigen trisaccharide]-Sp1	524	88
**G0058**	Fuc-α-1,2-Gal-β-1,4-GlcNAc-β-[blood H antigen trisaccharide]-Sp1	295	77
**G0059**	Gal-α-1,3-(Fuc-α-1,2)-Gal-β-[blood B antigen trisaccharide]-Sp1	364	91
**G0060**	Fuc-α-1,2-Gal-β-1,3-GlcNAc-β-1,3-Gal-β-1,4-Glc-β [LNFP I]-Sp1	597	151
**G0061**	Fuc-α-1,2-Gal-β-1,4-Glc-β-[blood H antigen trisaccharide]-Sp1	282	58
**G0062**	Gal-α-1,3-(Fuc-α-1,2)-Gal-β-1,4-Glc-β-[blood B antigen tetrasaccharide]-Sp1	189	30
**G0063**	(Fuc-α-1,2)-Gal-β-1,4-(Fuc-α-1,3)-GlcNAc-β-[Lewis Y]-Sp1	288	60
**G0064**	(Fuc-α-1,2)-Gal-β-1,3-(Fuc-α-1,4)-GlcNAc-β-[Lewis B]-Sp1	137	8
**G0065**	Gal-β-1,3-(Fuc-α-1,4)-GlcNAc-β-1,3-Gal-β-1,4-(Fuc-α-1,4)-Glc-β-[Lewis A]-Sp1	160	33
**G0066**	Gal-β-1,3-GalNAc-β-Sp1	109	16
**G0067**	Gal-β-1,3-(Neu5Ac-α-2,6)-GalNAc-β-Sp	308	31
**G0068**	Neu5Ac-α-2,6-Gal-β-1,3-GalNAc-β-Sp	444	105
**G0069**	Neu5Ac-α-2,6-Gal-β-1,3-(Neu5Ac-α-2,6)-GalNAc-β-Sp	302	84
**G0070**	Neu5Ac-α-2,3-Gal-β-1,3-(Neu5Ac-α-2,6)-GalNAc-β-Sp	237	29
**G0071**	Neu5Ac-α-2,6-(Neu5Ac-α-2,3)-Gal-β-1,3-GalNAc-β-Sp	286	93
**G0072**	GalNAc-β-1,4-(Neu5Ac-α-2,3)-Gal-β-1,4-Glc-β-[GM2]-Sp	315	18
**G0073**	GalNAc-β-1,4-(Neu5Ac-α-2,8-Neu5Ac-α-2,3)-Gal-β-1,4-Glc-β-[GD2]-Sp	16	1
**G0074**	Gal-α-1,4-Gal-β-1,4-GlcNAc-β-Sp1	330	46
**G0075**	β-D-Rha-Sp	409	106

**G0076**	Glc-α-1,4-Glc-β-Sp1	1033	111
**G0077**	Glc-α-1,6-Glc-α-1,4-Glc-β-Sp1	522	187
**G0078**	Maltotriose-β-Sp1	657	144
**G0079**	Glc-α-1,6-Glc-α-1,6-Glc-β-Sp1	628	121
**G0080**	Maltotetraose-β-Sp1	1227	119
**G0081**	GlcNAc-α-1,4-GlcA-β-1,4-GlcNAc-α1,4-GlcA-β-Sp	139	32
**G0082**	Maltohexaose-β-Sp1	8432	100
**G0083**	Maltoheptaose-β-Sp1	10,095	285
**G0084**	Acarbose-β-Sp1	570	83
**G0085**	D-Pentamannuronic acid-β-Sp1	447	81
**G0086**	L-Pentaguluronic acid-β-Sp1	610	205
**G0087**	D-Cellose-β-Sp1	929	280
**G0088**	Gal-α-1,3-Gal-β-Sp1	690	42
**G0089**	β-1,4-Xylotetrose-Sp1	262	46
**G0090**	Chitin-trisaccharide-Sp1	304	58
**G0091**	KDN-α-2,8-Neu5Ac-α-2,3-Gal-β-1,4-Glc-β-Sp	55	4
**G0092**	Neu5Ac-α-2,8-Neu5Gc-α-2,3-Gal-β-1,4-Glc-β-Sp	183	72
**G0093**	Neu5Ac-α-2,8-Neu5Ac-α-2,8-Neu5Ac-α-2,3-Gal-β-1,4-Glc-β-Sp3	64	10
**G0094**	Neu5Ac-a-2,8-Neu5Ac-a-2,6-Gal-b-1,4-Glc-Sp5	14	6
**G0095**	Gal-β-1,3-GalNAc-β-1,4-(Neu5Ac-α-2,3)-Gal-β-1,4-Glc-β-Sp1	157	30
**G0096**	Gentamicin sulfate	401	48
**G0097**	Kanamycin sulfate	773	201
**G0098**	Geneticin disulfate salt (G418)	156	4
**G0099**	Neomycin trisulfate	1094	305
**G0100**	SGP	184	57
**N010**	Man-α-1,6-(Man-α-1,3-)Man-α-1,6-(GlcNAc-β-1,2-Man-α-1,3-)Man-β-1,4-GlcNAc-β-1,4-GlcNAc-Sp5	145	38
**N011**	Man-α-1,6-(Man-α-1,3-)Man-α-1,6-(Gal-β-1,4-GlcNAc-β-1,2-Man-α-1,3-)Man-β-1,4-GlcNAc-β-1,4-GlcNAc-Sp5	106	13

**N012**	Man-α-1,6-(Man-α-1,3-)Man-α-1,6-(Neu5Ac-α-2,3-Gal-β-1,4-GlcNAc-β-1,2-Man-α-1,3-)Man-β-1,4-GlcNAc-β-1,4-GlcNAc-Sp5	86	13
**N013**	Man-α-1,6-(Man-α-1,3-)Man-α-1,6-(Neu5Ac-α-2,6-Gal-β-1,4-GlcNAc-β-1,2-Man-α-1,3-)Man-β-1,4-GlcNAc-β-1,4-GlcNAc-Sp5	95	16
**N014**	Man-α-1,6-(Man-α-1,3-)Man-α-1,6-[Gal-β-1,4-(Fuc-α-1,3-)GlcNAc-β-1,2-Man-α-1,3-]Man-β-1,4-GlcNAc-β-1,4-GlcNAc-Sp5	120	10
**N015**	Man-α-1,6-(Man-α-1,3-)Man-α-1,6-[Neu5Ac-α-2,3-Gal-β-1,4-(Fuc-α-1,3-)GlcNAc-β-1,2- Man-α-1,3-]Man-β-1,4-GlcNAc-β-1,4-GlcNAc-Sp5	117	5
**N020**	GlcNAc-β-1,2-Man-α-1,3-Man-β-1,4-GlcNAc-β-1,4-GlcNAc-Sp5	208	23
**N021**	Gal-β-1,4-GlcNAc-β-1,2-Man-α-1,3-Man-β-1,4-GlcNAc-β-1,4-GlcNAc-Sp5	144	12
**N022**	Neu5Ac-α-2,3-Gal-β-1,4-GlcNAc-β-1,2-Man-α-1,3-Man-β-1,4-GlcNAc-β-1,4-GlcNAc-Sp5	106	25
**N023**	Neu5Ac-α-2,6-Gal-β-1,4-GlcNAc-β-1,2-Man-α-1,3-Man-β-1,4-GlcNAc-β-1,4-GlcNAc-Sp5	127	16
**N024**	Gal-β-1,4-(Fuc-α-1,3-)GlcNAc-β-1,2-Man-α-1,3-Man-β-1,4-GlcNAc-β-1,4-GlcNAc-Sp5	113	3
**N025**	Neu5Ac-α-2,3-Gal-β-1,4-(Fuc-α-1,3-)GlcNAc-β-1,2-Man-α-1,3-Man-β-1,4-GlcNAc-β-1,4-GlcNAc-Sp5	86	9
**N026**	Gal-α-1,3-Gal-β-1,4-GlcNAc-β-1,2-Man-α-1,3-Man-β-1,4-GlcNAc-β-1,4-GlcNAc-Sp5	109	12
**N022G**	Neu5Gc-α-2,3-Gal-β-1,4-GlcNAc-β-1,2-Man-α-1,3-Man-β-1,4-GlcNAc-β-1,4-GlcNAc-Sp5	97	5
**N023G**	Neu5Gc-α-2,6-Gal-β-1,4-GlcNAc-β-1,2-Man-α-1,3-Man-β-1,4-GlcNAc-β-1,4-GlcNAc-Sp5	160	5
**N025G**	Neu5Gc-α-2,3-Gal-β-1,4-(Fuc-α-1,3-)GlcNAc-β-1,2-Man-α-1,3-Man-β-1,4-GlcNAc-β-1,4-GlcNAc-Sp5	92	10
**N030**	Man-α-1,6-(GlcNAc-β-1,2-Man-α-1,3-)Man-β-1,4-GlcNAc-β-1,4-GlcNAc-Sp5	170	8
**N210**	GlcNAc-β-1,2-Man-α-1,6-[GlcNAc(3Ac)-β-1,2-Man-α-1,3-Man-β-1,4-GlcNAc-β-1,4-GlcNAc-Sp5	105	15
**N040**	GlcNAc-β-1,2-Man-α-1,6-Man-β-1,4-GlcNAc-β-1,4-GlcNAc-Sp5	132	14
**N041**	Gal-β-1,4-GlcNAc-β-1,2-Man-α-1,3-Man-β-1,4-GlcNAc-β-1,4-GlcNAc-Sp5	212	26
**N042**	Neu5Ac-α-2,3-Gal-β-1,4-GlcNAc-β-1,2-Man-α-1,3-Man-β-1,4-GlcNAc-β-1,4-GlcNAc-Sp5	126	13
**N043**	Neu5Ac-α-2,6-Gal-β-1,4-GlcNAc-β-1,2-Man-α-1,3-Man-β-1,4-GlcNAc-β-1,4-GlcNAc-Sp5	132	1
**N044**	Gal-β-1,4-(Fuc-α-1,3-)GlcNAc-β-1,2-Man-α-1,3-Man-β-1,4-GlcNAc-β-1,4-GlcNAc-Sp5	163	19
**N045**	Neu5Ac-α-2,3-Gal-β-1,4-(Fuc-α-1,3-)GlcNAc-β-1,2-Man-α-1,3-Man-β-1,4-GlcNAc-β-1,4-GlcNAc-Sp5	156	10
**N050**	GlcNAc-β-1,2-Man-α-1,6-(Man-α-1,3-)Man-β-1,4-GlcNAc-β-1,4-GlcNAc-Sp5	324	59
**N051**	Gal-β-1,4-GlcNAc-β-1,2-Man-α-1,6-(Man-α-1,3-)Man-β-1,4-GlcNAc-β-1,4-GlcNAc-Sp5	287	38
**N052**	Neu5Ac-α-2,3-Gal-β-1,4-GlcNAc-β-1,2-Man-α-1,6-(Man-α-1,3-)Man-β-1,4-GlcNAc-β-1,4-GlcNAc-Sp5	155	10
**N053**	Neu5Ac-α-2,6-Gal-β-1,4-GlcNAc-β-1,2-Man-α-1,6-(Man-α-1,3-)Man-β-1,4-GlcNAc-β-1,4-GlcNAc-Sp	149	41

**N054**	Gal-β-1,4-(Fuc-α-1,3-)GlcNAc-β-1,2-Man-α-1,6-(Man-α-1,3-)Man-β-1,4-GlcNAc-β-1,4-GlcNAc-Sp5	225	16
**N055**	Neu5Ac-α-2,3-Gal-β-1,4-(Fuc-α-1,3-)GlcNAc-β-1,2-Man-α-1,6-(Man-α-1,3-)Man-β-1,4-GlcNAc-β-1,4-GlcNAc-Sp5	133	19
**TE001**	Neu5Ac-α-2,6-Gal-β-1,4-GlcNAc-Man-α-1,3-(Neu5Ac-α-2,6-Gal-β-1,4-GlcNAc-Man-α-1,6-)Man-β-1,4-GlcNAc-β-1,4-GlcNAc-β-Asn	14	0
**TE002**	Gal-β-1,4-GlcNAc-β-1,2-Man-α-1,3-(Gal-β-1,4-GlcNAc-β-1,2-Man-α-1,6-)Man-β-1,4-GlcNAc-β-1,4-GlcNAc-β-Asn	6	2
**TE003**	Neu5Gc-α-2,6-Gal-β-1,4-GlcNAc-β-1,2-Man-α-1,3-(Neu5Gc-α-2,6-Gal-β-1,4-GlcNAc-β-1,2-Man-α-1,6-)Man-β-1,4-GlcNAc-β-1,4-GlcNAc-β-Asn	12	3
**TE004**	Neu5Ac-α-2,3-Gal-β-1,4-GlcNAc-β-1,2-Man-α-1,3-(Neu5Ac-α-2,3-Gal-β-1,4-GlcNAc-β-1,2-Man-α-1,6-)Man-β-1,4-GlcNAc-β-1,4-GlcNAc-β-Asn	34	4
**TE005**	Neu5Gc-α-2,3-Gal-β-1,4-GlcNAc-β-1,2-Man-α-1,3-(Neu5Gc-α-2,3-Gal-β-1,4-GlcNAc-β-1,2-Man-α-1,6-)Man-β-1,4-GlcNAc-β-1,4-GlcNAc-β-Asn	37	11
**TE006**	Gal-β-1,4-(Fucα-1,3-)GlcNAc-β-1,2-Man-α-1,3-[Gal-β-1,4-(Fucα-1,3-)GlcNAc-β-1,2-Man-α-1,6-]Man-β-1,4-GlcNAc-β-1,4-GlcNAc-β-Asn	12	3
**TE007**	Gal-α-1,3-Gal-β-1,4-GlcNAc-β-1,2-Man-α-1,3-(Gal-α-1,3-Gal-β-1,4-GlcNAc-β-1,2-Man-α-1,6-)Man-β-1,4-GlcNAc-β-1,4-GlcNAc-β-Asn	8	1
**TE008**	Gal-β-1,4-(Fucα-1,3-)GlcNAc-β-1,2-Man-α-1,3-[Gal-β-1,4-(Fucα-1,3-)GlcNAc-β-1,2-Man-α-1,6-]Man-β-1,4-GlcNAc-β-1,4-GlcNAc-β-Asn	15	5
**TE009**	Neu5Ac-α-2,8-Neu5Ac-α-2,6-Gal-β-1,4-GlcNAc-β-1,2-Man-α-1,3-(Neu5Ac-α-2,8-Neu5Ac-α-2,6-Gal-β-1,4-GlcNAc-β-1,2-Man-α-1,6-)Man-β-1,4-GlcNAc-β-1,4-GlcNAc-β-Asn	4	4
**TE010**	Neu5Gc-α-2,8-Neu5Ac-α-2,6-Gal-β-1,4-GlcNAc-β-1,2-Man-α-1,3-(Neu5Gc-α-2,8-Neu5Ac-α-2,6-Gal-β-1,4-GlcNAc-β-1,2-Man-α-1,6-)Man-β-1,4-GlcNAc-β-1,4-GlcNAc-β-Asn	9	0
**TE011**	Neu5Ac-α-2,8-Neu5Gc-α-2,6-Gal-β-1,4-GlcNAc-β-1,2-Man-α-1,3-(Neu5Ac-α-2,8-Neu5Gc-α-2,6-Gal-β-1,4-GlcNAc-β-1,2-Man-α-1,6-)Man-β-1,4-GlcNAc-β-1,4-GlcNAc-β-Asn	8	2
**TE012**	Neu5Gc-α-2,8-Neu5Gc-α-2,6-Gal-β-1,4-GlcNAc-β-1,2-Man-α-1,3-(Neu5Gc-α-2,8-Neu5Gc-α-2,6-Gal-β-1,4-GlcNAc-β-1,2-Man-α-1,6-)Man-β-1,4-GlcNAc-β-1,4-GlcNAc-β-Asn	32	4
**TE013**	Neu5Ac-α-2,8-Neu5Ac-α-2,3-Gal-β-1,4-GlcNAc-β-1,2-Man-α-1,3-(Neu5Ac-α-2,8-Neu5Ac-α-2,3-Gal-β-1,4-GlcNAc-β-1,2-Man-α-1,6-)Man-β-1,4-GlcNAc-β-1,4-GlcNAc-β-Asn	11	2
**TE014**	Neu5Gc-α-2,8-Neu5Ac-α-2,3-Gal-β-1,4-GlcNAc-β-1,2-Man-α-1,3-(Neu5Gc-α-2,8-Neu5Ac-α-2,3-Gal-β-1,4-GlcNAc-β-1,2-Man-α-1,6-)Man-β-1,4-GlcNAc-β-1,4-GlcNAc-β-Asn	9	7
**TE015**	Neu5Ac-α-2,8-Neu5Gc-α-2,3-Gal-β-1,4-GlcNAc-β-1,2-Man-α-1,3-(Neu5Ac-α-2,8-Neu5Gc-α-2,3-Gal-β-1,4-GlcNAc-β-1,2-Man-α-1,6-)Man-β-1,4-GlcNAc-β-1,4-GlcNAc-β-Asn	1	0
**TE016**	Neu5Gc-α-2,8-Neu5Gc-α-2,3-Gal-β-1,4-GlcNAc-β-1,2-Man-α-1,3-(Neu5Gc-α-2,8-Neu5Gc-α-2,3-Gal-β-1,4-GlcNAc-β-1,2-Man-α-1,6-)Man-β-1,4-GlcNAc-β-1,4-GlcNAc-β-Asn	3	0
**TE017**	Neu5Ac-α-2,3-Gal-β-1,4-(Fucα-1,3-)GlcNAc-β-1,2-Man-α-1,3-[Neu5Ac-α-2,3-Gal-β-1,4-(Fucα-1,3-)GlcNAc-β-1,2-Man-α-1,6-]Man-β-1,4-GlcNAc-β-1,4-GlcNAc-β-Asn	9	1
**TE018**	Neu5Gc-α-2,3-Gal-β-1,4-(Fucα-1,3-)GlcNAc-β-1,2-Man-α-1,3-[Neu5Gc-α-2,3-Gal-β-1,4-(Fucα-1,3-)GlcNAc-β-1,2-Man-α-1,6-]Man-β-1,4-GlcNAc-β-1,4-GlcNAc-β-Asn	2	1

**TE019**	Neu5Ac-α-2,3-Gal-β-1,4-GlcNAc-β-1,3-Gal-β-1,4-GlcNAc-β-1,2-Man-α-1,3-(Neu5Ac-α-2,3-Gal-β-1,4-GlcNAc-β-1,3-Gal-β-1,4-GlcNAc-β-1,2-Man-α-1,6-)Man-β-1,4-GlcNAc-β-1,4-GlcNAc-β-Asn	6	7
**TE020**	Neu5Gc-α-2,3-Gal-β-1,4-GlcNAc-β-1,3-Gal-β-1,4-GlcNAc-β-1,2-Man-α-1,3-(Neu5Gc-α-2,3-Gal-β-1,4-GlcNAc-β-1,3-Gal-β-1,4-GlcNAc-β-1,2-Man-α-1,6-)Man-β-1,4-GlcNAc-β-1,4-GlcNAc-β-Asn	2	1
**TE021**	Neu5Ac-α-2,6-Gal-β-1,4-GlcNAc-β-1,3-(Neu5AC-α-2,6-)Gal-β-1,4-GlcNAc-β-1,2-Man-α-1,3-[Neu5Ac-α-2,6-Gal-β-1,4-GlcNAc-β-1,3-(Neu5AC-α-2,6-)Gal-β-1,4-GlcNAc-β-1,2-Man-α-1,6-]Man-β-1,4-GlcNAc-β-1,4-GlcNAc-β-Asn	1	0
**TE022**	Neu5Gc-α-2,6-Gal-β-1,4-GlcNAc-β-1,3-(Neu5GC-α-2,6-)Gal-β-1,4-GlcNAc-β-1,2-Man-α-1,3-[Neu5Gc-α-2,6-Gal-β-1,4-GlcNAc-β-1,3-(Neu5GC-α-2,6-)Gal-β-1,4-GlcNAc-β-1,2-Man-α-1,6-]Man-β-1,4-GlcNAc-β-1,4-GlcNAc-β-Asn	1	0
**TE023**	Neu5Ac-α-2,3-Gal-β-1,4-GlcNAc-β-1,3-(Neu5AC-α-2,6-)Gal-β-1,4-GlcNAc-β-1,2-Man-α-1,3-[Neu5Ac-α-2,3-Gal-β-1,4-GlcNAc-β-1,3-(Neu5AC-α-2,6-)Gal-β-1,4-GlcNAc-β-1,2-Man-α-1,6-]Man-β-1,4-GlcNAc-β-1,4-GlcNAc-β-Asn	25	2
**TE024**	GlcNAc-β-1,3-Gal-β-1,4-GlcNAc-β-1,2-Man-α-1,3-(GlcNAc-β-1,3-Gal-β-1,4-GlcNAc-β-1,2-Man-α-1,6-)Man-β-1,4-GlcNAc-β-1,4-GlcNAc-β-Asn	10	3
**TE025**	Gal-β-1,4-GlcNAc-β-1,3-Gal-β-1,4-GlcNAc-β-1,2-Man-α-1,3-(Gal-β-1,4-GlcNAc-β-1,3-Gal-β-1,4-GlcNAc-β-1,2-Man-α-1,6-)Man-β-1,4-GlcNAc-β-1,4-GlcNAc-β-Asn	7	1
**TE026**	GlcNAc-β-1,3-Gal-β-1,4-GlcNAc-β-1,3-Gal-β-1,4-GlcNAc-β-1,2-Man-α-1,3-(GlcNAc-β-1,3-Gal-β-1,4-GlcNAc-β-1,3-Gal-β-1,4-GlcNAc-β-1,2-Man-α-1,6-)Man-β-1,4-GlcNAc-β-1,4-GlcNAc-β-Asn	36	4
**TE027**	Gal-β-1,4-GlcNAc-β-1,3-Gal-β-1,4-GlcNAc-β-1,3-Gal-β-1,4-GlcNAc-β-1,2-Man-α-1,3-(Gal-β-1,4-GlcNAc-β-1,3-Gal-β-1,4-GlcNAc-β-1,3-Gal-β-1,4-GlcNAc-β-1,2-Man-α-1,6-)Man-β-1,4-GlcNAc-β-1,4-GlcNAc-β-Asn	7	9
**TE028**	GlcNAc-β-1,3-Gal-β-1,4-GlcNAc-β-1,3-Gal-β-1,4-GlcNAc-β-1,3-Gal-β-1,4-GlcNAc-β-1,2-Man-α-1,3-(GlcNAc-β-1,3-Gal-β-1,4-GlcNAc-β-1,3-Gal-β-1,4-GlcNAc-β-1,3-Gal-β-1,4-GlcNAc-β-1,2-Man-α-1,6-)Man-β-1,4-GlcNAc-β-1,4-GlcNAc-β-Asn	8	8
**TE029**	Gal-β-1,4-GlcNAc-β-1,3-Gal-β-1,4-GlcNAc-β-1,3-Gal-β-1,4-GlcNAc-β-1,3-Gal-β-1,4-GlcNAc-β-1,2-Man-α-1,3-(Gal-β-1,4-GlcNAc-β-1,3-Gal-β-1,4-GlcNAc-β-1,3-Gal-β-1,4-GlcNAc-β-1,3-Gal-β-1,4-GlcNAc-β-1,2-Man-α-1,6-)Man-β-1,4-GlcNAc-β-1,4-GlcNAc-β-Asn	4	1
**TE030**	GlcNAc-β-1,3-Gal-β-1,4-GlcNAc-β-1,3-Gal-β-1,4-GlcNAc-β-1,3-Gal-β-1,4-GlcNAc-β-1,3-Gal-β-1,4-GlcNAc-β-1,2-Man-α-1,3-(GlcNAc-β-1,3-Gal-β-1,4-GlcNAc-β-1,3-Gal-β-1,4-GlcNAc-β-1,3-Gal-β-1,4-GlcNAc-β-1,3-Gal-β-1,4-GlcNAc-β-1,2-Man-α-1,6-)Man-β-1,4-GlcNAc-β-1,4-GlcNAc-β-Asn	4	4
**TE031**	Gal-β-1,4-GlcNAc-β-1,3-Gal-β-1,4-GlcNAc-β-1,3-Gal-β-1,4-GlcNAc-β-1,3-Gal-β-1,4-GlcNAc-β-1,3-Gal-β-1,4-GlcNAc-β-1,2-Man-α-1,3-(Gal-β-1,4-GlcNAc-β-1,3-Gal-β-1,4-GlcNAc-β-1,3-Gal-β-1,4-GlcNAc-β-1,3-Gal-β-1,4-GlcNAc-β-1,3-Gal-β-1,4-GlcNAc-β-1,2-Man-α-1,6-)Man-β-1,4-GlcNAc-β-1,4-GlcNAc-β-Asn	3	1
**TE032**	GlcNAc-β-1,3-Gal-β-1,4-(Fucα-1,3-)GlcNAc-β-1,2-Man-α-1,3-[GlcNAc-β-1,3-Gal-β-1,4-(Fucα-1,3-)GlcNAc-β-1,2-Man-α-1,6-]Man-β-1,4-GlcNAc-β-1,4-GlcNAc-β-Asn	56	5
**TE033**	Gal-β-1,4-GlcNAc-β-1,3-Gal-β-1,4-(Fucα-1,3-)GlcNAc-β-1,2-Man-α-1,3-[Gal-β-1,4-GlcNAc-β-1,3-Gal-β-1,4-(Fucα-1,3-)GlcNAc-β-1,2-Man-α-1,6-]Man-β-1,4-GlcNAc-β-1,4-GlcNAc-β-Asn	5	4
**TE034**	Neu5Ac-α-2,3-Gal-β-1,4-GlcNAc-β-1,3-Gal-β-1,4-(Fucα-1,3-)GlcNAc-β-1,2-Man-α-1,3-[Neu5Ac-α-2,3-Gal-β-1,4-GlcNAc-β-1,3-Gal-β-1,4-(Fucα-1,3-)GlcNAc-β-1,2-Man-α-1,6-]Man-β-1,4-GlcNAc-β-1,4-GlcNAc-β-Asn	1	0
**TE035**	GlcNAc-β-1,3-Gal-β-1,4-GlcNAc-β-1,3-Gal-β-1,4-(Fucα-1,3-)GlcNAc-β-1,2-Man-α-1,3-[GlcNAc-β-1,3-Gal-β-1,4-GlcNAc-β-1,3-Gal-β-1,4-(Fucα-1,3-)GlcNAc-β-1,2-Man-α-1,6-]Man-β-1,4-GlcNAc-β-1,4-GlcNAc-β-Asn	16	10
**TE036**	Gal-β-1,4-GlcNAc-β-1,3-Gal-β-1,4-GlcNAc-β-1,3-Gal-β-1,4-(Fucα-1,3-)GlcNAc-β-1,2-Man-α-1,3-[Gal-β-1,4-GlcNAc-β-1,3-Gal-β-1,4-GlcNAc-β-1,3-Gal-β-1,4-(Fucα-1,3-)GlcNAc-β-1,2-Man-α-1,6-]Man-β-1,4-GlcNAc-β-1,4-GlcNAc-β-Asn	1	0

**TE037**	GlcNAc-β-1,3-Gal-β-1,4-GlcNAc-β-1,3-Gal-β-1,4-GlcNAc-β-1,3-Gal-β-1,4-(Fucα-1,3-)GlcNAc-β-1,2-Man-α-1,3-[GlcNAc-β-1,3-Gal-β-1,4-GlcNAc-β-1,3-Gal-β-1,4-GlcNAc-β-1,3-Gal-β-1,4-(Fucα-1,3-)GlcNAc-β-1,2-Man-α-1,6-]Man-β-1,4-GlcNAc-β-1,4-GlcNAc-β-Asn	5	3
**TE038**	Gal-β-1,4-GlcNAc-β-1,3-Gal-β-1,4-GlcNAc-β-1,3-Gal-β-1,4-GlcNAc-β-1,3-Gal-β-1,4-(Fucα-1,3-)GlcNAc-β-1,2-Man-α-1,3-[Gal-β-1,4-GlcNAc-β-1,3-Gal-β-1,4-GlcNAc-β-1,3-Gal-β-1,4-GlcNAc-β-1,3-Gal-β-1,4-(Fucα-1,3-)GlcNAc-β-1,2-Man-α-1,6-]Man-β-1,4-GlcNAc-β-1,4-GlcNAc-β-Asn	5	6
**TE039**	Gal-β-1,4-(Fucα-1,3-)GlcNAc-β-1,3-Gal-β-1,4-(Fucα-1,3-)GlcNAc-β-1,2-Man-α-1,3-[Gal-β-1,4-(Fucα-1,3-)GlcNAc-β-1,3-Gal-β-1,4-(Fucα-1,3-)GlcNAc-β-1,2-Man-α-1,6-]Man-β-1,4-GlcNAc-β-1,4-GlcNAc-β-Asn	2	1
**TE040**	GlcNAc-β-1,3-Gal-β-1,4-(Fucα-1,3-)GlcNAc-β-1,3-Gal-β-1,4-(Fucα-1,3-)GlcNAc-β-1,2-Man-α-1,3-[GlcNAc-β-1,3-Gal-β-1,4-(Fucα-1,3-)GlcNAc-β-1,3-Gal-β-1,4-(Fucα-1,3-)GlcNAc-β-1,2-Man-α-1,6-]Man-β-1,4-GlcNAc-β-1,4-GlcNAc-β-Asn	92	11
**TE041**	Gal-β-1,4-GlcNAc-β-1,3-Gal-β-1,4-(Fucα-1,3-)GlcNAc-β-1,3-Gal-β-1,4-(Fucα-1,3-)GlcNAc-β-1,2-Man-α-1,3-[Gal-β-1,4-GlcNAc-β-1,3-Gal-β-1,4-(Fucα-1,3-)GlcNAc-β-1,3-Gal-β-1,4-(Fucα-1,3-)GlcNAc-β-1,2-Man-α-1,6-]Man-β-1,4-GlcNAc-β-1,4-GlcNAc-β-Asn	19	0
**TE042**	Gal-β-1,4-(Fucα-1,3-)GlcNAc-β-1,3-Gal-β-1,4-(Fucα-1,3-)GlcNAc-β-1,3-Gal-β-1,4-(Fucα-1,3-)GlcNAc-β-1,2-Man-α-1,3-[Gal-β-1,4-(Fucα-1,3-)GlcNAc-β-1,3-Gal-β-1,4-(Fucα-1,3-)GlcNAc-β-1,3-Gal-β-1,4-(Fucα-1,3-)GlcNAc-β-1,2-Man-α-1,6-]Man-β-1,4-GlcNAc-β-1,4-GlcNAc-β-Asn	2	1
**TE043**	Gal-α-1,3-Gal-β-1,4-GlcNAc-β-1,3-Gal-β-1,4-GlcNAc-β-1,2-Man-α-1,3-(Gal-α-1,3-Gal-β-1,4-GlcNAc-β-1,3-Gal-β-1,4-GlcNAc-β-1,2-Man-α-1,6-)Man-β-1,4-GlcNAc-β-1,4-GlcNAc-β-Asn	3	0
**TE044**	Gal-α-1,3-Gal-β-1,4-GlcNAc-β-1,3-Gal-β-1,4-(Fucα-1,3-)GlcNAc-β-1,2-Man-α-1,3-[Gal-α-1,3-Gal-β-1,4-GlcNAc-β-1,3-Gal-β-1,4-(Fucα-1,3-)GlcNAc-β-1,2-Man-α-1,6-]Man-β-1,4-GlcNAc-β-1,4-GlcNAc-β-Asn	23	4
**TE045**	Gal-α-1,3-Gal-β-1,4-(Fucα-1,3-)GlcNAc-β-1,3-Gal-β-1,4-(Fucα-1,3-)GlcNAc-β-1,2-Man-α-1,3-[Gal-α-1,3-Gal-β-1,4-(Fucα-1,3-)GlcNAc-β-1,3-Gal-β-1,4-(Fucα-1,3-)GlcNAc-β-1,2-Man-α-1,6-]Man-β-1,4-GlcNAc-β-1,4-GlcNAc-β-Asn	52	18
**TE046**	Gal-α-1,3-Gal-β-1,4-GlcNAc-β-1,3-Gal-β-1,4-GlcNAc-β-1,3-Gal-β-1,4-GlcNAc-β-1,2-Man-α-1,3-(Gal-α-1,3-Gal-β-1,4-GlcNAc-β-1,3-Gal-β-1,4-GlcNAc-β-1,3-Gal-β-1,4-GlcNAc-β-1,2-Man-α-1,6-)Man-β-1,4-GlcNAc-β-1,4-GlcNAc-β-Asn	7	1
**TE047**	Gal-α-1,3-Gal-β-1,4-GlcNAc-β-1,3-Gal-β-1,4-GlcNAc-β-1,3-Gal-β-1,4-(Fucα-1,3-)GlcNAc-β-1,2-Man-α-1,3-[Gal-α-1,3-Gal-β-1,4-GlcNAc-β-1,3-Gal-β-1,4-GlcNAc-β-1,3-Gal-β-1,4-(Fucα-1,3-)GlcNAc-β-1,2-Man-α-1,6-]Man-β-1,4-GlcNAc-β-1,4-GlcNAc-β-Asn	22	18
**TE048**	Gal-α-1,3-Gal-β-1,4-GlcNAc-β-1,3-Gal-β-1,4-(Fucα-1,3-)GlcNAc-β-1,3-Gal-β-1,4-(Fucα-1,3-)GlcNAc-β-1,2-Man-α-1,3-[Gal-α-1,3-Gal-β-1,4-GlcNAc-β-1,3-Gal-β-1,4-(Fucα-1,3-)GlcNAc-β-1,3-Gal-β-1,4-(Fucα-1,3-)GlcNAc-β-1,2-Man-α-1,6-]Man-β-1,4-GlcNAc-β-1,4-GlcNAc-β-Asn	19	9
**TE049**	Gal-α-1,3-Gal-β-1,4-(Fucα-1,3-)GlcNAc-β-1,3-Gal-β-1,4-(Fucα-1,3-)GlcNAc-β-1,3-Gal-β-1,4-(Fucα-1,3-)GlcNAc-β-1,2-Man-α-1,3-[Gal-α-1,3-Gal-β-1,4-(Fucα-1,3-)GlcNAc-β-1,3-Gal-β-1,4-(Fucα-1,3-)GlcNAc-β-1,3-Gal-β-1,4-(Fucα-1,3-)GlcNAc-β-1,2-Man-α-1,6-]Man-β-1,4-GlcNAc-β-1,4-GlcNAc-β-Asn	71	6
**TE050**	Gal-α-1,3-Gal-β-1,4-GlcNAc-β-1,3-Gal-β-1,4-GlcNAc-β-1,3-Gal-β-1,4-GlcNAc-β-1,3-Gal-β-1,4-GlcNAc-β-1,2-Man-α-1,3-(Gal-α-1,3-Gal-β-1,4-GlcNAc-β-1,3-Gal-β-1,4-GlcNAc-β-1,3-Gal-β-1,4-GlcNAc-β-1,3-Gal-β-1,4-GlcNAc-β-1,2-Man-α-1,6-)Man-β-1,4-GlcNAc-β-1,4-GlcNAc-β-Asn	1	0
**H0100**	GlcNAc-β-1,3-(GlcNAc-β-1,6-)Gal-β-1,4-Glc-Sp5	198	41
**H0101**	Gal-β-1,4-GlcNAc-β-1,3-(Gal-β-1,4-GlcNAc-β-1,6-)Gal-β-1,4-Glc-Sp5	102	25

**H0103**	Neu5Gc-α-2,6-Gal-β-1,4-GlcNAc-β-1,3-(Neu5Gc-α-2,6-Gal-β-1,4-GlcNAc-β-1,6-)Gal-β-1,4-Glc-Sp5	87	5
**H0105**	Fuc-α-1,2-Gal-β-1,4-GlcNAc-β-1,3-(Fuc-α-1,2-Gal-β-1,4-GlcNAc-β-1,6-)Gal-β-1,4-Glc-Sp5	85	5
**H0106**	Fuc-α-1,2-Gal-β-1,4-(Fuc-α-1,3-)GlcNAc-β-1,3-[Fuc-α-1,2-Gal-β-1,4-(Fuc-α-1,3-)GlcNAc-β-1,6-]Gal-β-1,4-Glc-Sp5	69	6
**H0200**	Gal-β-1,4-GlcNAc-β-1,3-(GlcNAc-β-1,6-)Gal-β-1,4-Glc-Sp5	98	27
**H0201**	Neu5Ac-α-2,6-Gal-β-1,4-GlcNAc-β-1,3-(GlcNAc-β-1,6-)Gal-β-1,4-Glc-Sp5	109	15
**H0202**	Neu5Gc-α-2,6-Gal-β-1,4-GlcNAc-β-1,3-(GlcNAc-β-1,6-)Gal-β-1,4-Glc-Sp5	125	15
**H0203**	Gal-β-1,4-(Fuc-α-1,3-)GlcNAc-β-1,3-(GlcNAc-β-1,6-)Gal-β-1,4-Glc-Sp5	91	16
**H0204**	Fuc-α-1,2-Gal-β-1,4-GlcNAc-β-1,3-(GlcNAc-β-1,6-)Gal-β-1,4-Glc-Sp5	97	2
**H0205**	Neu5Ac-α-2,6-Gal-β-1,4-GlcNAc-β-1,3-(Gal-β-1,4-GlcNAc-β-1,6-)Gal-β-1,4-Glc-Sp5	90	1
**H0207**	Gal-β-1,4-(Fuc-α-1,3-)GlcNAc-β-1,3-(Gal-β-1,4-GlcNAc-β-1,6-)Gal-β-1,4-Glc-Sp5	112	23
**H0208**	Fuc-α-1,2-Gal-β-1,4-GlcNAc-β-1,3-(Gal-β-1,4-GlcNAc-β-1,6-)Gal-β-1,4-Glc-Sp5	144	5
**H0209**	Fuc-α-1,2-Gal-β-1,4-(Fuc-α-1,3-)GlcNAc-β-1,3-(GlcNAc-β-1,6-)Gal-β-1,4-Glc-Sp5	131	13
**H0210**	Fuc-α-1,2-Gal-β-1,4-(Fuc-α-1,3-)GlcNAc-β-1,3-[Gal-β-1,4-(Fuc-α-1,3-)GlcNAc-β-1,6-]Gal-β-1,4-Glc-Sp5	148	3
**H0300**	GlcNAc-β-1,3-(Gal-β-1,4-GlcNAc-β-1,6-)Gal-β-1,4-Glc-Sp5	124	13
**H0301**	GlcNAc-β-1,3-(Neu5Ac-α-2,6-Gal-β-1,4-GlcNAc-β-1,6-)Gal-β-1,4-Glc-Sp5	104	11
**H0303**	GlcNAc-β-1,3-[Gal-β-1,4-(Fuc-α-1,3-)GlcNAc-β-1,6-]Gal-β-1,4-Glc-Sp5	100	3
**H0304**	Fuc-α-1,2-GlcNAc-β-1,3-(Gal-β-1,4-GlcNAc-β-1,6-)Gal-β-1,4-Glc-Sp5	100	9
**H0305**	Gal-β-1,4-GlcNAc-β-1,3-(Neu5Ac-α-2,6-Gal-β-1,4-GlcNAc-β-1,6-)Gal-β-1,4-Glc-Sp5	70	2
**H0306**	Gal-β-1,4-GlcNAc-β-1,3-(Neu5Gc-α-2,6-Gal-β-1,4-GlcNAc-β-1,6-)Gal-β-1,4-Glc-Sp5	71	15
**H0307**	Gal-β-1,4-GlcNAc-β-1,3-[Gal-β-1,4-(Fuc-α-1,3-)GlcNAc-β-1,6-]Gal-β-1,4-Glc-Sp5	104	15
**H0400**	Gal-β-1,4-Glc-Sp	879	178
**H0402**	GalNAc-β-1,3-Gal-β-1,4-Glc-Sp	204	2
**H0403**	Neu5Ac-α-2,3-Gal-β-1,4-Glc-Sp	404	11
**H0404**	Neu5Gc-α-2,3-Gal-β-1,4-Glc-Sp	316	23
**H0405**	Neu5Ac-α-2,6-Gal-β-1,4-Glc-Sp	335	43
**H0406**	Neu5Gc-α-2,6-Gal-β-1,4-Glc-Sp	365	64
**H0407**	Gal-α-1,3-Gal-β-1,4-Glc-Sp	246	8

**H0408**	Neu5Ac-α-2,8-Neu5Ac-α-2,3-Gal-β-1,4-Glc-Sp	47	12
**H0409**	Neu5Ac-α-2,8-Neu5Ac-α-2,6-Gal-β-1,4-Glc-Sp	109	22
**H0410**	Neu5Ac-α-2,3-Gal-α-1,3-Gal-β-1,4-Glc-Sp	195	16
**H0411**	Neu5Ac-α-2,6-Gal-α-1,3-Gal-β-1,4-Glc-Sp	239	43
**H0500**	Gal-α-1,4-Gal-β-1,4-Glc-Sp5	210	2
**H0503**	GalNAc-β-1,3-Gal-α-1,4-Gal-β-1,4-Glc-Sp5	128	20
**H0504**	Gal-β-1,3-GalNAc-β-1,3-Gal-α-1,4-Gal-β-1,4-Glc-Sp5	95	5
**H0505**	Fuc-α-1,2-Gal-β-1,3-GalNAc-β-1,3-Gal-α-1,4-Gal-β-1,4-Glc-Sp5	78	2
**H0600**	Gal-β-1,4-GlcNAc-β-1,3-Gal-β-1,4-Glc-Sp5	99	22
**H0601**	Gal-β-1,4-(Fuc-α-1,3-)GlcNAc-β-1,3-Gal-β-1,4-Glc-Sp5	153	19
**H0602**	Fuc-α-1,2-Gal-β-1,4-GlcNAc-β-1,3-Gal-β-1,4-Glc-Sp5	127	13
**H0603**	GlcNAc-β-1,3-Gal-β-1,4-GlcNAc-β-1,3-Gal-β-1,4-Glc-Sp5	133	10
**H0604**	Neu5Ac-α-2,3-Gal-β-1,4-GlcNAc-β-1,3-Gal-β-1,4-Glc-Sp5	105	14
**H0606**	Gal-β-1,4-GlcNAc-β-1,3-(Neu5Ac-α-2,6-)Gal-β-1,4-Glc-Sp5	131	19
**H0608**	Fuc-α-1,2-Gal-β-1,4-(Fuc-α-1,3-)GlcNAc-β-1,3-Gal-β-1,4-Glc-Sp5	151	1
**H0609**	Neu5Ac-α-2,3-Gal-β-1,4-(Fuc-α-1,3-)GlcNAc-β-1,3-Gal-β-1,4-Glc-Sp5	146	11
**H0610**	GlcNAc-β-1,3-Gal-β-1,4-(Fuc-α-1,3-)GlcNAc-β-1,3-Gal-β-1,4-Glc-Sp5	263	5
**H0700**	Gal-β-1,4-GlcNAc-β-1,3-Gal-β-1,4-GlcNAc-β-1,3-Gal-β-1,4-Glc-Sp5	90	7
**H0701**	GlcNAc-β-1,3-Gal-β-1,4-GlcNAc-β-1,3-Gal-β-1,4-GlcNAc-β-1,3-Gal-β-1,4-Glc-Sp5	140	13
**H0800**	Gal-β-1,4-GlcNAc-β-1,3-Gal-β-1,4-GlcNAc-β-1,3-Gal-β-1,4-GlcNAc-β-1,3-Gal-β-1,4-Glc-Sp5	90	0
**H0900**	Gal-β-1,3-GlcNAc-β-1,3-(GlcNAc-β-1,6-)Gal-β-1,4-Glc-Sp5	199	1
**L1001**	Neu5Ac-α-2,3-Gal-β-1,4-Glc-Sp5	83	10
**L1002**	Neu5Gc-α-2,3-Gal-β-1,4-Glc-Sp5	126	11
**L1003**	Kdn-α-2,3-Gal-β-1,4-Glc-Sp5	126	4
**L1011**	Neu5Ac-a-2,3-(GalNAc-b-1,4-)Gal-b-1,4-Glc-Sp5	48	8
**L1012**	Neu5Gc-a-2,3-(GalNAc-b-1,4-)Gal-b-1,4-Glc-Sp5	68	10
**L1013**	Kdn-a-2,3-(GalNAc-b-1,4-)Gal-b-1,4-Glc-Sp5	55	14

**L1021**	Neu5Ac-a-2,3-(Gal-b-1,3-GalNAc-b-1,4-)Gal-b-1,4-Glc-Sp5	50	7
**L1022**	Neu5Gc-a-2,3-(Gal-b-1,3-GalNAc-b-1,4-)Gal-b-1,4-Glc-Sp5	79	7
**L1023**	Kdn-a-2,3-(Gal-b-1,3-GalNAc-b-1,4-)Gal-b-1,4-Glc-Sp5	154	7
**L1201**	Neu5Ac-α-2,8-Neu5Ac-α-2,3-Gal-β-1,4-Glc-Sp5	58	13
**L1202**	Neu5Gc-α-2,8-Neu5Ac-α-2,3-Gal-β-1,4-Glc-Sp5	88	2
**L1203**	Kdn-α-2,8-Neu5Ac-α-2,3-Gal-β-1,4-Glc-Sp5	77	17
**L1204**	Neu5Ac-α-2,8-Neu5Gc-α-2,3-Gal-β-1,4-Glc-Sp5	47	9
**L1205**	Neu5Gc-α-2,8-Neu5Gc-α-2,3-Gal-β-1,4-Glc-Sp5	43	1
**L1206**	Kdn-α-2,8-Neu5Gc-α-2-,3-Gal-β-1,4-Glc-Sp5	25	13
**L1207**	Neu5Ac-α-2,8-Kdn-α-2,3-Gal-β-1,4-Glc-Sp5	35	4
**L1209**	Kdn-α-2,8-Kdn-α-2,3-Gal-β-1,4-Glc-Sp5	54	3
**L1211**	Neu5Ac-α-2,8-Neu5Ac-α-2,3-(GalNAc-b-1,4-)Gal-β-1,4-Glc-Sp5	20	7
**L1212**	Neu5Gc-α-2,8-Neu5Ac-α-2,3-(GalNAc-b-1,4-)Gal-β-1,4-Glc-Sp5	23	4
**L1213**	Kdn-α-2,8-Neu5Ac-α-2,3-(GalNAc-b-1,4-)Gal-β-1,4-Glc-Sp5	28	2
**L1214**	Neu5Ac-α-2,8-Neu5Gc-α-2,3-(GalNAc-b-1,4-)Gal-β-1,4-Glc-Sp5	25	12
**L1215**	Neu5Gc-α-2,8-Neu5Gc-α-2,3-(GalNAc-b-1,4-)Gal-β-1,4-Glc-Sp5	22	1
**L1216**	Kdn-α-2,8-Neu5Gc-α-2,3-(GalNAc-b-1,4-)Gal-β-1,4-Glc-Sp5	21	10
**L1221**	Neu5Ac-α-2,8-Neu5Ac-α-2,3-(Gal-b-1,3-GalNAc-b-1,4-)Gal-β-1,4-Glc-Sp5	17	3
**L1222**	Neu5Gc-α-2,8-Neu5Ac-α-2,3-(Gal-b-1,3-GalNAc-b-1,4-)Gal-β-1,4-Glc-Sp5	33	3
**L1225**	Neu5Gc-α-2,8-Neu5Gc-α-2,3-(Gal-b-1,3-GalNAc-b-1,4-)Gal-β-1,4-Glc-Sp5	45	3
**L1226**	Kdn-α-2,8-Neu5Gc-α-2,3-(Gal-b-1,3-GalNAc-b-1,4-)Gal-β-1,4-Glc-Sp5	66	9
**L2000**	GlcNAc-β-1,3-Gal-β-1,4-Glc-Sp5	398	6
**L2100**	Gal-β-1,4-GlcNAc-β-1,3-Gal-β-1,4-Glc-Sp5	121	12
**L2101**	Gal-a-1,3-Gal-β-1,4-GlcNAc-β-1,3-Gal-β-1,4-Glc-Sp5	90	8
**L2102**	Gal-a-1,4-Gal-β-1,4-GlcNAc-β-1,3-Gal-β-1,4-Glc-Sp5	120	18
**L2111**	Neu5Ac-a-2,3-Gal-β-1,4-GlcNAc-β-1,3-Gal-β-1,4-Glc-Sp5	84	4
**L2112**	Neu5Gc-a-2,3-Gal-β-1,4-GlcNAc-β-1,3-Gal-β-1,4-Glc-Sp5	20	1

**L2113**	Kdn-a-2,3-Gal-β-1,4-GlcNAc-β-1,3-Gal-β-1,4-Glc-Sp5	62	5
**L2121**	Neu5Ac-a-2,8-Neu5Ac-a-2,3-Gal-β-1,4-GlcNAc-β-1,3-Gal-β-1,4-Glc-Sp5	20	15
**L2122**	Neu5Gc-a-2,8-Neu5Ac-a-2,3-Gal-β-1,4-GlcNAc-β-1,3-Gal-β-1,4-Glc-Sp5	59	9
**L2103**	Gal-β-1,4-(Fuc-α-1,3-)GlcNAc-β-1,3-Gal-β-1,4-Glc-Sp5	105	30
**L2104**	Gal-a-1,3-Gal-β-1,4-(Fuc-α-1,3-)GlcNAc-β-1,3-Gal-β-1,4-Glc-Sp5	119	0
**L2131**	Neu5Ac-a-2,3-Gal-β-1,4-(Fuc-α-1,3-)GlcNAc-β-1,3-Gal-β-1,4-Glc-Sp5	97	12
**L2132**	Neu5Gc-a-2,3-Gal-β-1,4-(Fuc-α-1,3-)GlcNAc-β-1,3-Gal-β-1,4-Glc-Sp5	76	1
**L2133**	Kdn-a-2,3-Gal-β-1,4-(Fuc-α-1,3-)GlcNAc-β-1,3-Gal-β-1,4-Glc-Sp5	92	1
**L2191**		86	20
**L2192**		64	7
**L2200**	GlcNAc-β-1,3-Gal-β-1,4-GlcNAc-β-1,3-Gal-β-1,4-Glc-Sp5	96	9
**L2300**	Gal-β-1,4-GlcNAc-β-1,3-Gal-β-1,4-GlcNAc-β-1,3-Gal-β-1,4-Glc-Sp5	75	5
**L2301**	Gal-a-1,3-Gal-β-1,4-GlcNAc-β-1,3-Gal-β-1,4-GlcNAc-β-1,3-Gal-β-1,4-Glc-Sp5	63	0
**L2302**	Gal-a-1,4-Gal-β-1,4-GlcNAc-β-1,3-Gal-β-1,4-GlcNAc-β-1,3-Gal-β-1,4-Glc-Sp5	69	10
**L2311**	Neu5Ac-a-2,3-Gal-β-1,4-GlcNAc-β-1,3-Gal-β-1,4-GlcNAc-β-1,3-Gal-β-1,4-Glc-Sp5	50	7
**L2312**	Neu5Gc-a-2,3-Gal-β-1,4-GlcNAc-β-1,3-Gal-β-1,4-GlcNAc-β-1,3-Gal-β-1,4-Glc-Sp5	69	6
**L2303**	Gal-β-1,4-GlcNAc-β-1,3-Gal-β-1,4-(Fuc-α-1,3-)GlcNAc-β-1,3-Gal-β-1,4-Glc-Sp5	68	4
**L2304**	Gal-β-1,4-(Fuc-α-1,3-)GlcNAc-β-1,3-Gal-β-1,4-(Fuc-α-1,3-)GlcNAc-β-1,3-Gal-β-1,4-Glc-Sp5	74	7
**L2391**		70	4
**L2392**		76	5
**L2900**	Gal-β-1,3-GlcNAc-β-1,3-Gal-β-1,4-Glc-Sp5	282	29
**L2911**	Neu5Ac-a-2,3-Gal-β-1,3-GlcNAc-β-1,3-Gal-β-1,4-Glc-Sp5	116	9
**L2912**	Neu5Gc-a-2,3-Gal-β-1,3-GlcNAc-β-1,3-Gal-β-1,4-Glc-Sp5	120	25
**L2913**	Kdn-a-2,3-Gal-β-1,3-GlcNAc-β-1,3-Gal-β-1,4-Glc-Sp5	104	15
**L3100**	Gal-α-1,4-Gal-β-1,4-Glc-Sp5	113	4
**L3101**	GalNAc-β-1,3-Gal-α-1,4-Gal-β-1,4-Glc-Sp5	78	18
**L3102**	Gal-β-1,3-GalNAc-β-1,3-Gal-α-1,4-Gal-β-1,4-Glc-Sp5	87	29

**L3111**	Neu5Ac-a-2,3-Gal-β-1,3-GalNAc-β-1,3-Gal-α-1,4-Gal-β-1,4-Glc-Sp5	44	12
**L3112**	Neu5Gc-a-2,3-Gal-β-1,3-GalNAc-β-1,3-Gal-α-1,4-Gal-β-1,4-Glc-Sp5	55	2
**L3113**	Kdn-a-2,3-Gal-β-1,3-GalNAc-β-1,3-Gal-α-1,4-Gal-β-1,4-Glc-Sp5	75	8
**L3103**		84	4
**L3200**	Gal-α-1,3-Gal-β-1,4-Glc-Sp5	111	2
**L3201**	GalNAc-β-1,3-Gal-α-1,3-Gal-β-1,4-Glc-Sp5	79	10
**L3202**	Gal-β-1,3-GalNAc-β-1,3-Gal-α-1,3-Gal-β-1,4-Glc-Sp5	70	13
**L3211**	Neu5Ac-a-2,3-Gal-β-1,3-GalNAc-β-1,3-Gal-α-1,3-Gal-β-1,4-Glc-Sp5	65	5
**L3212**	Neu5Gc-a-2,3-Gal-β-1,3-GalNAc-β-1,3-Gal-α-1,3-Gal-β-1,4-Glc-Sp5	67	9
**L3213**	Kdn-a-2,3-Gal-β-1,3-GalNAc-β-1,3-Gal-α-1,3-Gal-β-1,4-Glc-Sp5	71	3
